# Comparison of multiple interventions for older adults with Alzheimer disease or mild cognitive impairment: A PRISMA-compliant network meta-analysis

**DOI:** 10.1097/MD.0000000000010744

**Published:** 2018-05-18

**Authors:** Jing-hong Liang, Yong Xu, Lu Lin, Rui-xia Jia, Hong-bo Zhang, Lei Hang

**Affiliations:** a Department of Child Health, Jiangsu Key Laboratory of Preventive and Translational Medicine for Geriatric Diseases, School of Public Health, Soochow University; b School of Nursing, Medical College of Soochow University, Suzhou, PR China.

**Keywords:** Alzheimer disease, cognitive interventions, mild cognitive impairment, network meta-analysis

## Abstract

**Background:**

The increasing prevalence of Alzheimer disease (AD) emphasizes the need for effective treatments. Both pharmacological therapies such as nutrition therapy (NT) and nonpharmacologic therapies including traditional treatment or personalized treatment (e.g., physical exercise, music therapy, computerized cognitive training) have been approved for the treatment of AD or mild cognitive impairment (MCI) in numerous areas.

**Methods:**

The aim of this study was to compare 4 types of interventions, physical exercise (PE), music therapy (MT), computerized cognitive training (CCT), and NT, in older adults with mild to moderate AD or MCI and identify the most effective intervention for their cognitive function. We used a system of search strategies to identify relevant studies and include randomized controlled trials (RCTs), placebo-controlled trials evaluating the efficacy and safety of 4 interventions in patients with AD or MCI. We updated the relevant studies which were published before March 2017 as a full-text article. Using Bayesian network meta-analysis (NMA), we ranked cognitive ability based objectively on Mini-Mental State Examination (MMSE), and assessed neuropsychiatric symptoms based on Neuropsychiatric Inventory (NPI). Pairwise and network meta-analyses were sequentially performed for efficacy and safety of intervention compared to control group through RCTs included.

**Results:**

We included 17 RCTs. Fifteen trials (n = 1747) were pooled for cognition and no obvious heterogeneity was found (*I*^2^ = 21.7%, *P* = .212) in NMA, the mean difference (MD) of PE (MD = 2.1, confidence interval [CI]: 0.44–3.8) revealed that PE was significantly efficacious in the treatment group in terms of MMSE. Five trials (n = 660) assessed neuropsychiatric symptoms with an obvious heterogeneity (*I*^2^ = 61.6%, *P* = .034), the MD of CCT (MD = −7.7, CI: −14 to −2.4), revealing that CCT was significantly efficacious in NPI.

**Conclusions:**

As the first NMA comparing different interventions for AD and MCI, our study suggests that PE and CCT might have a significant improvement in cognition and neuropsychiatric symptoms respectively. Moreover, nonpharmacological therapies might be better than pharmacological therapies.

## 1 Introduction

Alzheimer disease (AD) is a neurological degenerative disease that would obtain progressive development but concealed in the early days. Clinically characterized by memory impairment, aphasia, disability, visual impairment, executive dysfunction, and personality and behavioral changes,^[1,2]^ patients living with AD have a poor self-living ability and impose a series of burden on their family, caregivers, health-care system even society. As a significant potential risk factor for AD, MCI is generally considered a precursor to AD.^[3]^ Although there was a large amount of objective evidence that MCI patients have experienced a decline in cognitive function, their abilities in activities of daily living (ADL) are still functional.^[4]^

The world's older population currently comprises nearly 900 million people, most of which come from relatively poor countries. Nowadays, more than 46 million people around the world suffer from AD, and by 2050 the number is estimated to reach 131.5 million. The total estimated worldwide cost of dementia is US $818 billion.^[5]^ Recently, a study showed that the risk of AD is increasing in men and women as their age increases, but more prevalent in women (rate per 100 person-years = 2.50 (1.85–3.41)) than in men (rate per 100 person-years = 1.89 (1.22–2.94)).^[6]^ Several organizations, such as the National Institute for Health and Care Excellence, suggest that management of patients with AD should be tailored to their needs. The organizations of AD focus on retarding the progressive cognitive dysfunctions, maintaining functional status, improving quality of life, minimizing adverse events (AEs), modulating caregiver stress, and relieving the economic burden of the family.

Pharmacological therapies consist of multifarious cognitive enhancers and it is still not clear whether they are the optimal treatment for AD. Moreover, evidence established by several studies has strongly shown that the use of some specialist drugs like cholinesterase inhibitors increases the risk of AEs in patients with AD. For example, cardiac medications like β-blockers may increase risk of bradycardia, and antiinflammatories may increase risk for gastrointestinal bleeding.^[7]^

Nonpharmacological therapies have attracted considerable attention as a safe, relatively inexpensive and scalable intervention that aims to maintain cognition in patients with AD and mild cognitive impairment (MCI), which include social support, daily activities, personalized cognitive treatment, advanced technical assistance, and support from the caregivers. Nonpharmacological cognitive interventions for AD and MCI include physical exercise (PE) and music therapy (MT), as well as computerized cognitive training (CCT), an efficacious and intelligently cognitive intervention. Several randomized, controlled trials (RCTs) have assessed the efficacy and safety of nonpharmacological therapies (PE, MT, CCT)^[8–27]^ or pharmacological therapies (NT)^[28–33]^ compared with control group (CG). However, the sample size of the previous studies was too small. In addition, no direct comparisons between cognitive interventions have been made. Generally speaking, almost no study have tried to answer such a sharp and debatable question—how to choose an optimal therapy from these interventions to treat older adults with AD or MCI. In the absence of direct evidence and large sample size, recently, a promising but much controversial extension of meta-analysis, network meta-analysis (NMA), has been increasingly used.

As the extension of traditional meta-analyses, NMA can simultaneously compare at least 2 interventions and pool data from different trials. It also enhances the relative effectiveness of inference for each intervention through direct and indirect information.^[34,35]^ Transitivity assumption is the pivotal assumption in NMA, which requires the balance of the distribution of potential effect modifiers across the treatment comparisons.^[36–38]^

NMA is helpful when investigators are interested in summarizing 2 or more of the treatment results and the hierarchy of these treatments. Although there are doubts about these methodological issues for sample size, relevant outcomes, and heterogeneity sources, but for more comparisons, NMA may obtain more accurate and reliable results than traditional meta-analysis.^[39]^ In this study, we employed this novel differential meta-analysis method to estimate the comparative efficacy and safety associated with cognitive interventions versus CG for AD or MCI. Our aim was to provide relatively effective and safe comparative evidence when identifying the optimal intervention for AD or MCI patients.

## 2 Methods

### 2.1 Literature search

PUBMED, EMBASE, and the Cochrane Central Register of Controlled Trials were used for preliminary literature search before March 2017. With a highly sensitive strategy, we identified relevant randomized controlled trials (RCTs). We used the MESH terms “Alzheimer's Disease, Cognitive Therapy, Physical Exercise, Music Therapy, Computer-Assisted, Nutrition therapy, randomized controlled trials” and keywords “Disease, Alzheimer” or “Alzheimer Dementia” or“ Alzheimer Type Dementia” or “ Alzheimer Type Senile Dementia” or “Cognitive Methods” or “Computer-Assisted Therapy” or “Computer Assisted Protocol Directed Therapy” or “Physical Activity” or “Aerobic Exercise” or “Exercise Trainings” or “Nutritional Support” to search for related literature.

Moreover, we additionally scanned the bibliography of the included studies, such as studies in reports and reference lists of identified studies from published meta-analyses. The search covered the full-text of the reports published before March 1, 2017. This NMA was prepared according to the preferred reporting items for systematic reviews and meta-analyses (PRISMA) guidelines.^[40]^ All analyses were based on previous published studies, thus no ethical approval and patient consent are required.

### 2.2 Eligibility criteria and data abstraction

We used population, interventions, comparisons, outcomes, study designs (PICOS) criteria. Eligible studies are RCTs that included older adults with AD or MCI and conducted a cognitive intervention compared with each other, or control group. The particular PICOS criteria are:

*Population*: Older adults with AD or MCI diagnosed using various criteria. (Individual research has different measurement method, but it does not affect the normal assessment of AD and MCI.)
*Interventions*: Cognitive interventions including physical exercise, music therapy, computerized cognitive training (nonpharmacological therapies), and nutrition therapy (pharmacological therapy).
*Comparisons*: Cognitive interventions, control group alone or in any combination.
*Outcomes*: The principal outcome was evaluated by validated assessment of MMSE, as the efficacy of cognitive interventions. As the second outcome, NPI was used to assess the neuropsychiatric symptoms. The above outcomes were employed by an adequate number of the included trials and thus our NMA can be conducted.
*Study design*: We confined to RCTs for they are the optimal standard for examining interventions. And we did not include other types of trials in our NMA.

Two authors (J-hL, H-bZ) independently identified and evaluated articles during the initial literature search according to the above criteria and extracted information into an electronic database. Appropriateness of group allocation, blinding, intended indication, population characteristics, specific interventions, and the completeness of outcome report. Titles and abstracts were screened firstly, and if the article was potentially relevant, full-text article was retrieved. Once any discrepancies emerged, the authors would discuss with each other, and the third author (LH) was asked to resolve the divergence if necessary. A unanimous agreement must be reached for these eligibility criteria by all authors.

Each study we extracted included demographic characteristics (e.g., gender, age mean and standard deviation, type of AD), study aims, treatment time, outcomes (e.g., ADAS-Cog, MMSE, NPI), and study areas. If reports were of the same trial at different follow-up periods, data of the last report were used for analysis. We used the mean, sample size and their standard deviation (SD) from each trial to analyze the group-specific of participants for continuous outcome.

### 2.3 Outcome measures

Different from traditional meta-analysis, our NMA did not extract the relevant outcome for each output, and only analyzed intersected outcomes reported in the original RCTs. The primary outcome was MMSE which evaluated the cognitive domain. Scores and cognitive ability are proportional. The secondary outcome was NPI, which assessed the neuropsychiatric symptoms. Scores and neuropsychiatric symptoms are inversely proportional. The means and SDs of the change from baseline were extracted. To ensure data precision, 2 authors (J-hL, H-bZ) independently extracted all of the data and discrepancies were settled by discussion or the involvement of a third author (LH). All authors were completely unanimous in selecting the outcome.

### 2.4 Statistical analysis

We first analyzed the summary data and demographic characteristics of each study. We also quantitatively estimated heterogeneity across studies with the help of *I*^2^ statistic^[41]^ (ranges from 0% to 100%, the higher the *I*^2^, the greater the heterogeneity), and looked at the funnel plots to evaluate obvious publication biases based on visual inspection, after which the NMA was conducted. The above random effects models in traditional meta-analysis was used to estimate variance between studies by using STATA, version 12 (Stata Corp, College Station, TX). The reason why we used the random effects model rather than the fixed effects model is that this might be the most appropriate and most conservative analysis of the variance between the studies.

As a natural extension of traditional meta-analysis for summarizing comparisons between treatment pairs,^[34]^ the random effect Bayesian statistical model was implemented to compare the indirect evidence for 4 cognitive interventions with placebo (cognitive interventions comparison: physical exercise vs musical therapy vs computerized cognitive training vs nutrition therapy vs placebo) combining all the descriptive data from various studies.^[34,35,38,42]^ In the Bayesian framework, all parameters are treated as random variables. For each incorporated parameter, its posterior distribution is estimated by placing the appropriate prior distribution using the Markov chain.^[43]^ The number of tuning iterations was set at 5000 and the number of simulation iterations at 20,000. The degree of convergence of the model was evaluated by visually inspecting the trace plot combined with density plot and the potential scale reduction factors.^[44]^ We extracted the mean and SD of the MMSE at the last observation of the studies, and computed the standardized mean change (Hedges’ adjusted g) from baseline as the gist of difference between the treatment groups. We also use the uniform method to evaluated the NPI scale as the measure of the neuropsychiatric symptoms of patients. For each summary statistic, a 95% credible interval (95% CI) was computed.

The probability that which intervention was the most efficacious intervention was derived from the proportion of the best ranking in all simulation operations.^[45]^ The Bayesian approach has a superiority of being able to provide the most effective cognitive intervention probability even if the standard method may determine that there is no significant difference between them. We used the network rank option to estimate the ranking probabilities. Probability values were summarized and reported as surface under the cumulative ranking (SUCRA).^[42]^ If the corresponding cognitive intervention of the SUCRA is always 1, it is ranked first and 0 if it always ranks last. We also analyzed relative rankings among each cognitive intervention (second, third, best, etc.), for some additional cases, the best cognitive intervention might be unavailable, more expensive, or contraindicated in some patients. Since our study only made the indirect comparison between each cognitive intervention, we could not calculate the difference of standardized mean differences (MDs) between direct and indirect comparisons to estimate the consistency of direct and indirect evidence.^[46]^ The above analyses were performed using Gemtc package (3.32 version) in R. At last, we used a slightly adapted version of the risk of bias approach of the Cochrane Collaboration to assess the quality of each included study,^[47]^ which performed in Review Manager (5.3 version).

## 3 Results

**Figure 1:**
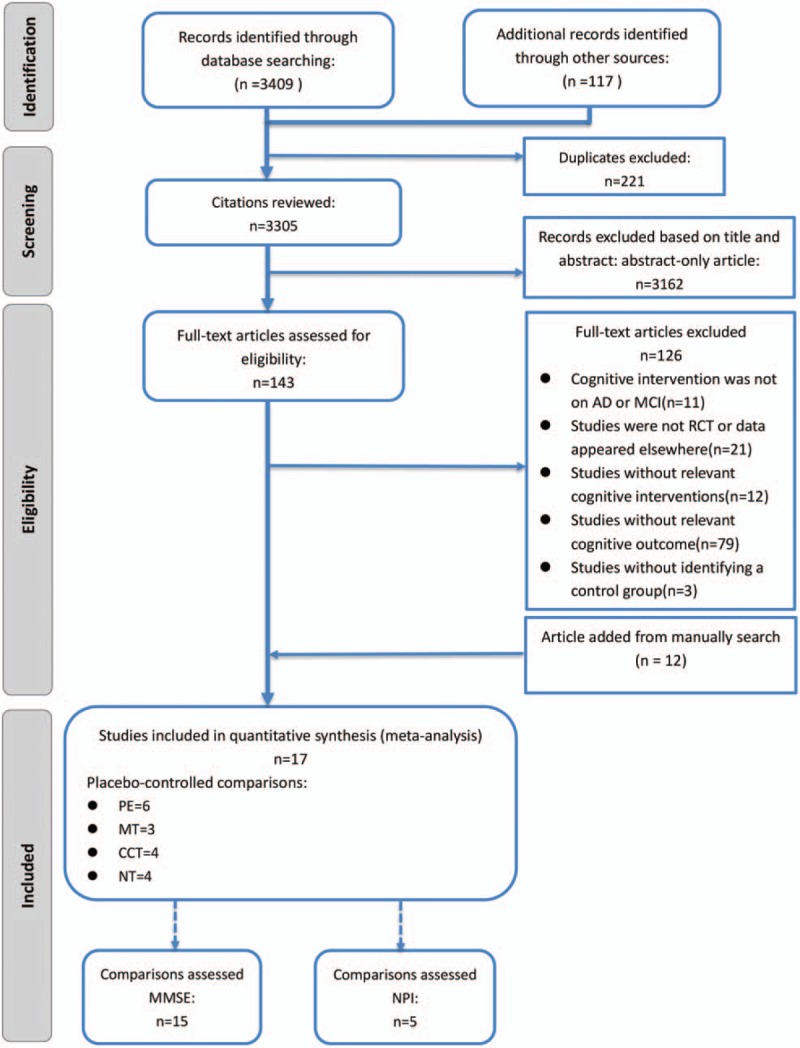
Literature review flowchart. AD = Alzheimer disease, ADAS-Cog = Alzheimer Disease Assessment Scale, cognitive subscale, CCT = computerized cognitive training, MCI = mild cognition impairment, MMSE = Mini-Mental State Examination, MT = music therapy, NPI = Neuropsychiatric Inventory, NT = nutrition therapy, PE = physical exercise, RCT = randomized controlled trial.

Figure 1 summarizes the selection process. We identified relevant studies for review of title and abstract at an initial screening. We used an extensive search strategy to retrieve the full texts of potentially eligible RCTs. It therefore seems unlikely that we missed some relevant trial.^[48]^ Three thousand five hundred twenty-six RCTs evaluating 4 different cognitive treatments were identified from initial screening, and 20 studies met our inclusion criteria, of which 17 studies were designed as RCTs and 3 studies were ruled out for having not identified a control group. All participating authors agreed on the methodology for selection and assessment. Fifteen studies assessed MMSE and 5 assessed NPI.

Table 1 presents the baseline data of demographic characteristics from 17 trials included. A total of 1931 AD patients underwent PIO (Population, Intervention, Outcomes) strategies. The trials were published between 2004 and 2016 and the majority of them were from US and Europe (N = 1748, 91%). Trials recruited participants mostly from their home. The mean age of all samples ranged from 69.8 to 86.1 years (one study lacked the data of baseline age).^[17]^ Fifty-five percent of the participants were women, and the average scores of MMSE for all samples ranged from 7.9 to 27.9 at baseline. At last, the average scores of NPI at baseline ranged from 5.0 to 18.7.

**Table 1:**
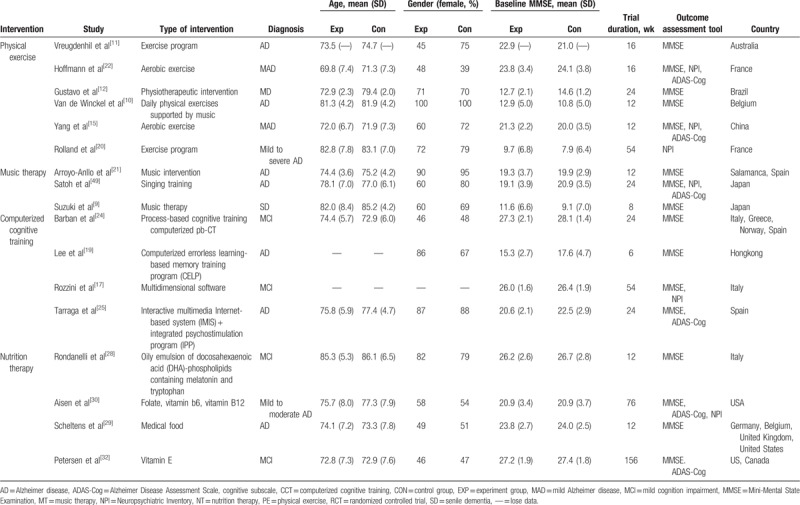
Baseline chart.

**Figure 2:**
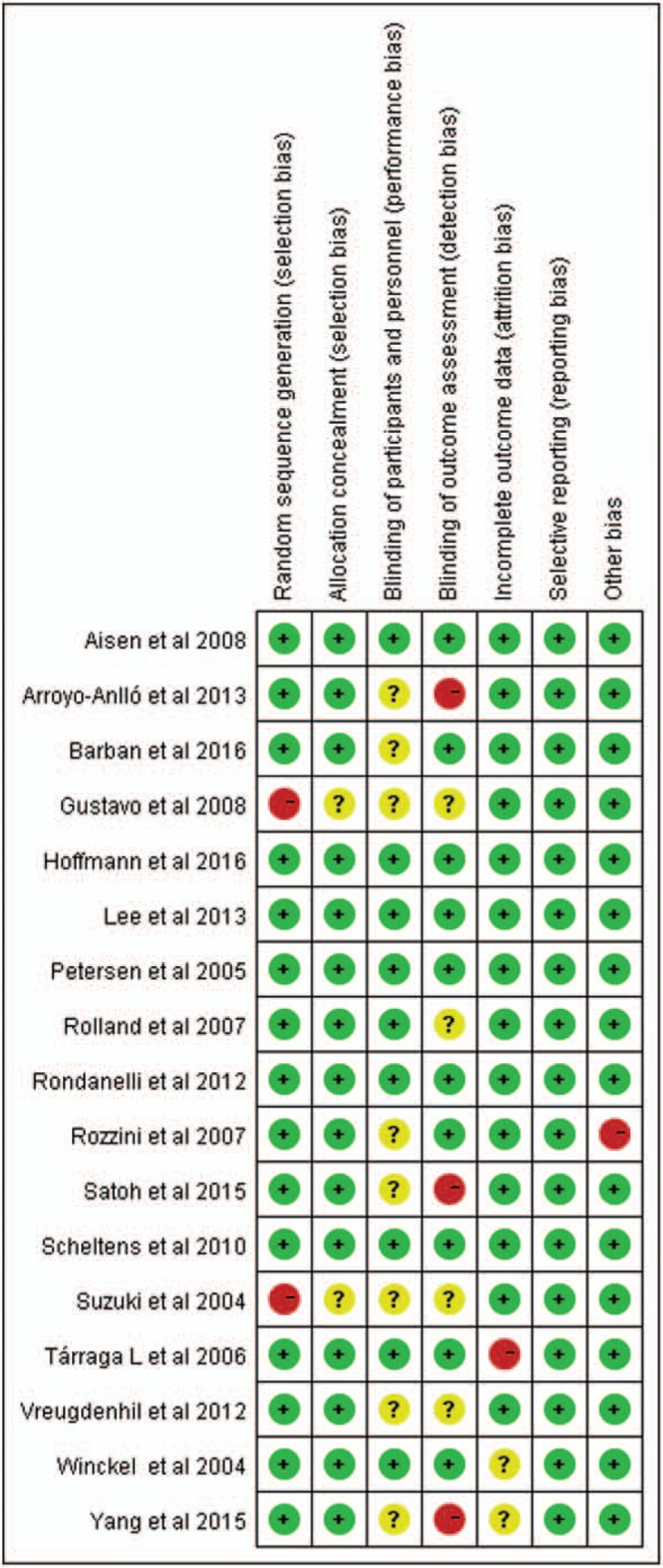
Risk of bias assessment.

**Figure 3:**
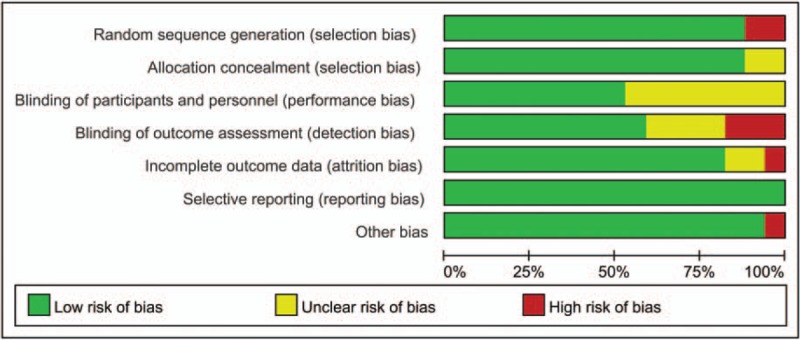
Risk of bias assessment (summary graph).

Figures 2 and 3 present the degree of risk of bias for all studies included. The vast majority of studies had a low risk of outcome data integrity. By contrast, the blinding of patient and investigator were unclear generally. The overall quality of the studies included in our study was modest.

### 3.1 Efficacy and ranking of treatment arm

#### 3.1.1 Primary outcome

**Figure 4:**
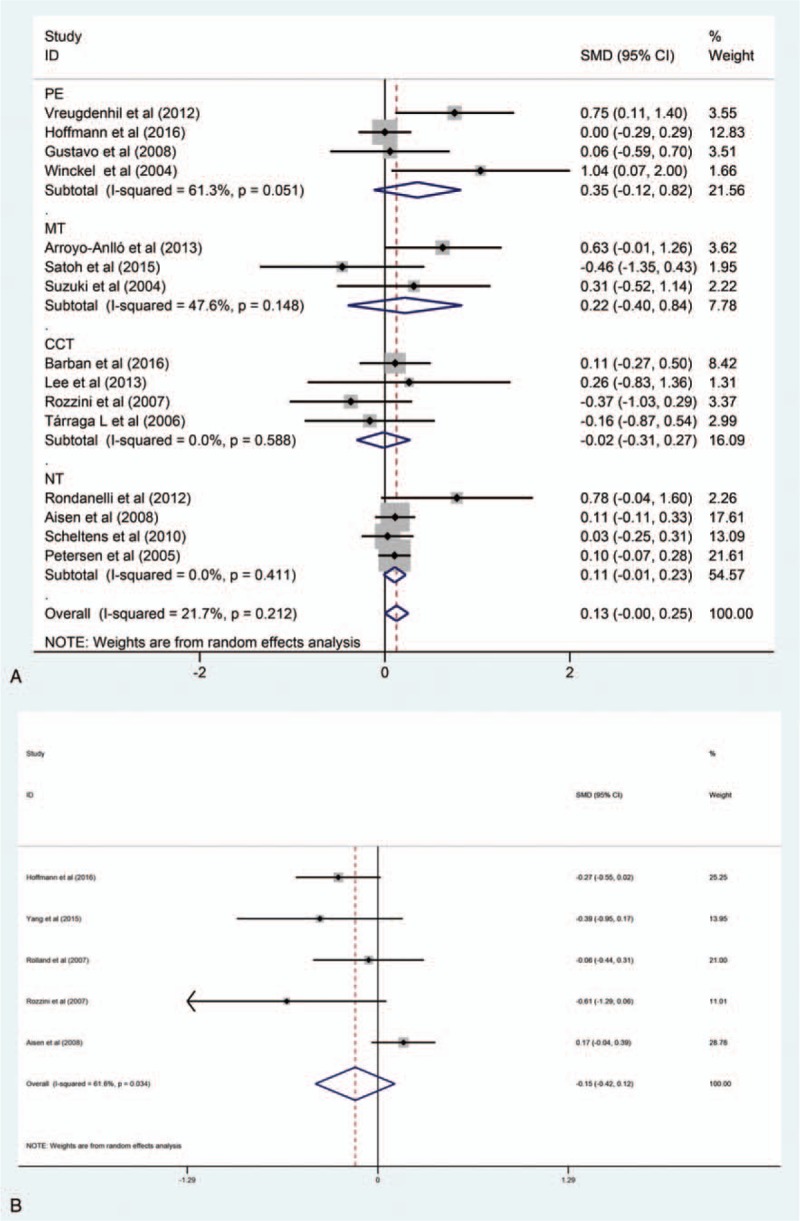
(A) The forest plot of primary outcome (summary graph). (B) The forest plot of secondary outcome (summary graph). CCT = computerized cognitive training, CG = control group, MT = music therapy, NT = nutrition therapy, PE = physical exercise, SMD = standard mean difference.

**Figure 5:**
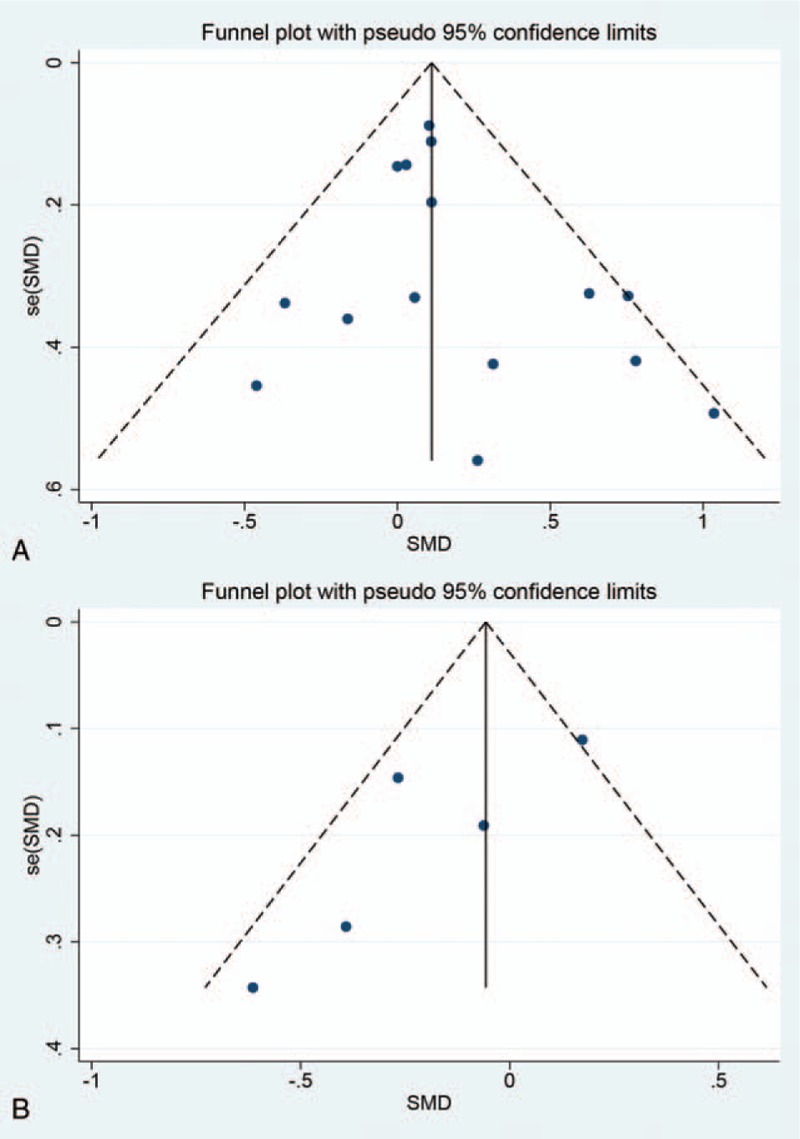
(A) Funnel plot of cognition. (B) Funnel plot of neuropsychiatric symptoms.

Among 17 studies included, 15 recorded relevant data about cognition, 4 for PE, 3 for MT, 4 for CCT, and 4 for NT. The absence of obvious heterogeneity (*I*^2^ = 21.7%, *P* = .212) was shown by preliminary meta-analysis (Fig. 4A). The funnel plot showed a symmetric distribution (Fig. 5A), indicating no hint of publication bias.

**Figure 6:**
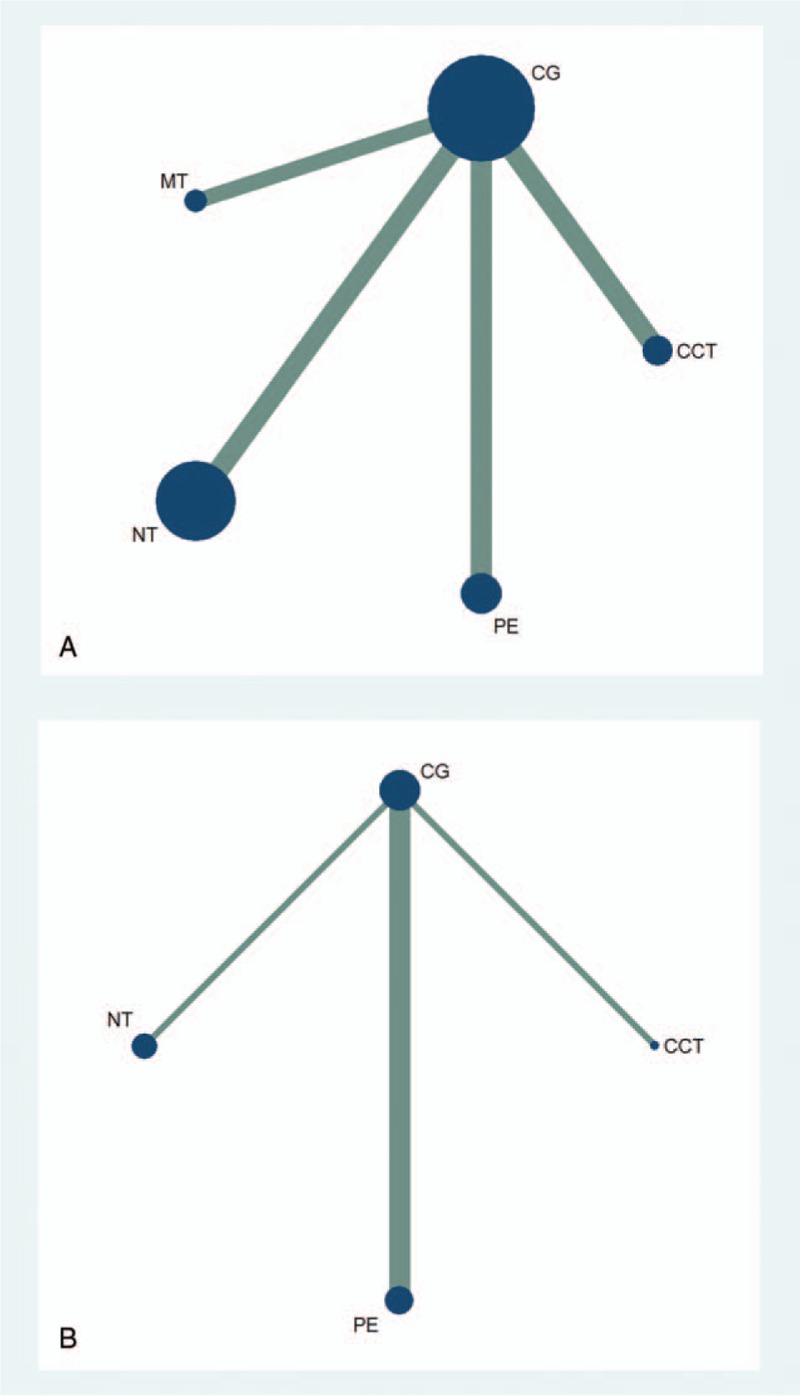
(A) Network of cognitive interventions comparison of cognition for the network meta-analysis. (B) Network of cognitive interventions comparison of neuropsychiatric symptoms for the network meta-analysis. CCT = computerized cognitive training, CG = control group, MT = music therapy, NT = nutrition therapy, PE = physical exercise.

**Figure 7:**
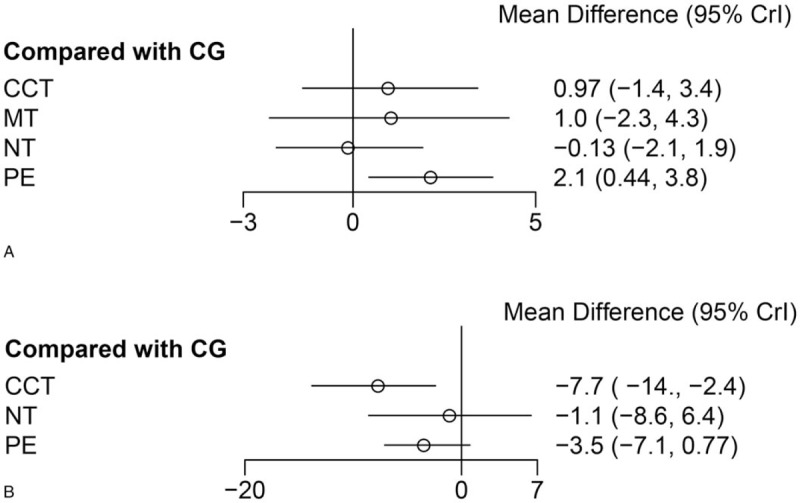
(A) Forest plot of cognition. (B) Forest plot of neuropsychiatric symptoms. CCT = computerized cognitive training, CG = control group, MT = music therapy, NT = nutrition therapy, PE = physical exercise.

**Figure 8:**
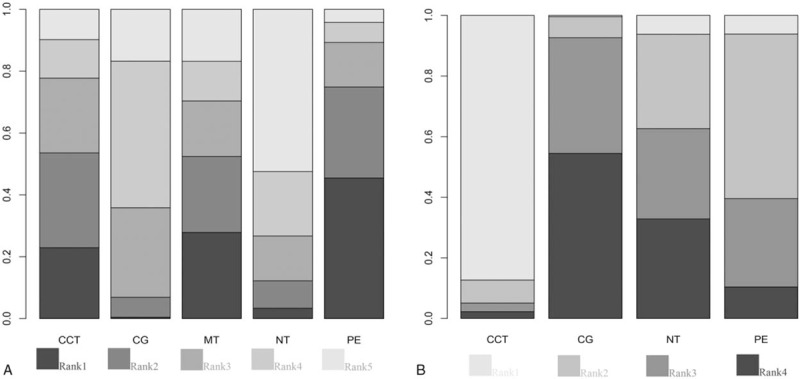
(A) Accumulate histogram of cognition. (B) Accumulate histogram of neuropsychiatric symptoms. CCT = computerized cognitive training, CG = control group, MT = music therapy, NT = nutrition therapy, PE = physical exercise.

At last, 15 eligible studies were finally included and reflected in the network relationship plot (Fig. 6A). Our analysis revealed that only PE had a significantly greater improvement than CG (Fig. 7A). The accumulate histogram (Fig. 8A) presents the probability of rank for each cognitive intervention, which indicated that PE was the highest in probability among all the 4 cognitive interventions (SUCRA = 0.45), followed by CCT (SUCRA = 0.30), and MT (SUCRA = 0.17). In contrast, NT seemed to have the lowest probability.

#### 3.1.2 Secondary outcome

*NPI*: Five studies reported relevant data about neuropsychiatric symptoms, 3 for PE, 1 for CCT, and 1 for NT. The presence of an obvious heterogeneity was shown by preliminary meta-analysis (*I*^2^ = 61.6%, *P* = .034) (Fig. 4B). Since only 5 studies contributed data, the meta-regression could not be conducted. The number of small studies we analyzed could further explain the potential source of the heterogeneity. And the funnel plots (Fig. 5B) showed a fairly symmetric distribution, indicating no hint of publication bias.

Figure 6B shows a network of 5 eligible studies. Our analysis revealed that only CCT had a significantly greater improvement than CG (Fig. 7B). The accumulate histogram (Fig. 8B) presents the probability of rank for each cognitive intervention, which indicated that CCT was the highest in probability among all the 4 cognitive interventions (SUCRA = 0.87), followed by PE (SUCRA = 0.54), and NT (SUCRA = 0.29).

## 4 Discussion

To the best of our knowledge, no previous study has solved the problem that which cognitive intervention is the relatively best intervention for AD or MCI. Therefore, as the first NMA of cognitive interventions for patients with AD or MCI in which indirect evidence was used appraising the relative effectiveness and safety of cognitive interventions across trials simultaneously, our study attempted to summarize available data to suggest that the highest probability of being the best intervention for cognitive decline and neuropsychiatric symptoms lies in PE (SUCAR = 0.45) and CCT (SUCAR = 0.87) respectively. The above findings will be reinforced by our analysis of previous meta-analyses.

We applied a trial sequential analysis to detect the robustness and reliability of evidence for relative effectiveness of each cognitive intervention. The trials in previous meta-analyses ^[50–59]^ (PUBMED search March 1, 2017) only investigated the efficacy and safety of PE, MT, CCT, and NT, respectively, and lacked a synthesized analysis among them. By contrast, our NMA assessed PE, MT, CCT and incorporated NT using 4 pairwise MDs. Integrating indirect comparisons in our NMA resulted in higher statistical precision in scientific comparisons of cognitive interventions against a control group. This integration makes the comparison of different interventions more explicit and facilitates interpretation. The potential correlations between these 4 MDs were accounted for in our NMA, and linking to modeling of indirect comparisons provided greater statistical power and more precise estimates.^[60–62]^ The totality of the evidence we extracted, largely based on trials in PE, MT, CCT, NT showed that further trials of cognitive interventions versus no cognitive interventions or control group are likely to have positive effect, except for some specific trials.^[30,32]^

Based on relative effect estimates and SUCAR, nowadays, PE seems to be the most effective cognitive intervention when we consider a cognitive therapy and CCT is the most effective cognitive intervention for neuropsychiatric symptoms. The cumulative probability ranking obtained through the Bayesian NMA cannot be considered as decisive conclusion because it was probably compromised by the lack of a significant difference among the cognitive interventions. For example, PE ranked the first in cognition but did not have superiority over any of the other cognitive interventions, which might be due to the fact that PE-relevant studies contributed a relatively greater deal of evidence in the network (6 out of 15 studies), and thus significant differences between these cognitive interventions were not found.

Previous studies have consistently demonstrated that almost all of these 4 cognitive interventions have beneficial effects on older adults with AD or MCI, PE in particular. Various kinds of moderate PE^[10–12,14–16,20,22,26,27]^ including “Walking program”, “Whole-body vibration”, “Treadmill training” had demonstrated that it was useful for AD and MCI through improvement in cognitive function or other areas. Some previous studies demonstrated low intensity or multiple exercise were able to improve neuropsychiatric symptoms in older adults with AD or MCI.^[63,64]^ moderate-to-high intensity PE can also improve cognitive ability.^[22]^ It seemed that PE as a relatively common intervention can effectively improve the core domains in patients of AD. However, NT is a double-edged sword, because it can improve the cognitive ability but at the same time may cause some AEs such as vomiting and diarrhea,^[29,30,32]^ which might explain why NT did not make an obvious improvement in cognitive ability of older adults with AD or MCI. By contrast, CCT as a relatively safe and inexpensive cognitive intervention has been increasingly applied. Some trials using a variety of computer-related advanced technologies to achieve CCT.^[17,18,25]^ It is worth mentioning that there are 2 meta-analyses of which the subjects were healthy older adults and which concluded that CCT were moderately effective in long-term improvement of cognition.^[65,66]^ Moreover, the majority of studies suggest that cognitive intervention is a long-term not temporary process.^[17,50,51,55]^ However, network meta-analyses synthesizes various cognitive interventions, and the measures of discrepancy between them are fairly obvious. This is probably the main reason for heterogeneity. NT is the only cognitive intervention we included which may cause AEs, which is why the probability rank of this intervention was low. Although previous meta-analyses^[50–59]^ provide high-quality evidence that PE, MT, CCT, NT can improve cognitive ability and quality of life in people with AD or MCI, but the trials they included only compared single cognitive intervention with only a control group. By contrast, our Bayesian network meta-analyses actualized the integration of different interventions. Since our results were based on indirectly randomized evidence, we were convinced that our study probably provides the best evidence of the efficacy and safety of these 4 cognitive interventions.

In summary, PE had the best effective improvement in cognitive ability and the second best in neuropsychiatric symptoms. CCT had the best result in improving neuropsychiatric symptoms and was relatively inexpensive. MT has a relatively low probability of being the best intervention for cognitive ability and neuropsychiatric symptoms. However, its safety factor and cost is relatively the best compared with other cognitive interventions. It should be noted the efficacy of a series of nondrug interventions to improve cognitive ability of AD and MCI patients have all been proven by research, such as estrogen replacement therapy,^[67,68]^ psychotherapy.^[69,70]^ But quite a number of trails were restricted by appropriate endpoints, which resulted in this individually cognitive interventions lack of the relevant endpoints whose efficacy we must adopt to analysis. Therefore, from our conclusion above, PE, MT, CCT all have beneficial effects on older adults with AD and MCI, especially PE^[58,71]^ and MT^[72,73]^ as relatively obtained easily interventions. AD and MCI are progressive neurodegenerative disorders, and are still incurable. Any cognitive intervention that could possibly slow down the progressive of AD and MCI patients, it worth disseminating. We may create an assumption that PE and MT as a potent, convenient, selective cognitive interventions were play a positive role in helping improve the cognitive function for older adults with AD or MCI.

### 4.1 Strengths and limitations

Rather than only grouping various interventions into CCT or human intervention, as the biggest strength, our NMA assessed each intervention individually and compared all major interventions simultaneously. Then, potential bias was reduced in the conduction of our review by having 2 independent authors (J-hL, H-bZ) scan through the search output, extract the relevant data, classify each intervention, and assess the methodological quality of each trial. We performed an extensive search strategy across several databases and sources to obtain an adequate number of eligible studies, with no language restriction. We also extensively searched the bibliographies of published studies. In addition, the cognitive intervention of CCT is complex and multifaceted and the number of relevant trials is very small, which proves the particular significance of our NMA.

From the methodological point of view, our NMA demonstrates a series of preponderance of Bayesian NMA for comparing various cognitive interventions and for evaluating the relative effectiveness and safety of multifarious interventions. In this context, the results of our NMA are likely to be more useful for decision makers, service commissioners and caregivers when they are making choices among different alternatives than results from multiple separate traditionally meta-analyses, because several relevant outcomes have been assessed simultaneously. It uses common random parameters to compare different interventions, which combines experimental evidence from indirectly randomized comparisons with observational evidence from adjusted indirect comparisons derived from trials.^[74]^

The limitations of our study also need to be acknowledged. Firstly, as the biggest limitation, the number of studies and the number of patients included in the study were relatively small. The studies included in our NMA used the same scale as the basis, the outcomes of which were presented as a continuous variable. In the analysis section, we extracted the mean, SD, and sample size values at baseline and at last observation for analysis. However, a few studies lost their data, which made the number of available studies even less. There are significant differences among cognitive interventions such as in the method section. The above-mentioned reasons explain why the number of our included studies was limited. In particular, the informative evidence of the direct comparisons between cognitive interventions was limited by the absence of relevant studies. Because no direct comparative trial was found through our search strategy, our study lacked direct evidence. No direct evidence was available when we performed NMA, and thus the evaluation of consistency could not be achieved. Secondly, only 5 of 17 studies included in the analyses were double-blind, and details of allocation were noted in 15 of 17 studies, indicating that publication bias and selective reporting biases could not be ruled out. Specific intervention regimens and patient populations varied across studies, which might cause heterogeneity. In addition, our study data were limited by the outcome of the intersection, a number of studies used their specific scales to present outcomes. And quite a number of trials were restricted by appropriate endpoints, which resulted in individual cognitive intervention lacking the relevant endpoints we must adopt, for example MT lack NPI data that only 5 studies included. That is the reason why we could not evaluate this intervention objectively in the end. Moreover, similar to previous traditional meta-analyses, our study yielded heterogeneity due to the small number of studies, although funnel plots did not suggest presence of heterogeneity and an extensive search strategy was used to identify relevant trials. The NMA is complex, and it is difficult for decision makers to explain the results. For example, under the accumulate histogram analysis of MMSE, PE was most likely to be the best cognitive intervention. This might be because the PE intervention had a relative larger sample size used for NMA. At last, we have not extracted the number of patients who have been observed in trials of numerous AEs or other reasons, which is because only three studies^[29,30,32]^ mentioned the AEs and we could not evaluate them in this analysis.

## 5 Conclusion

In conclusion, our NMA suggested that PE is the optimum cognitive intervention for patients with AD or MCI while CCT is the optimum one for neuropsychiatric symptoms. Relatively speaking, MT is the most safe intervention but its efficacy is moderate. And NT is the last choice to manage AD or MCI because of its 2-sidedness. The results of our NMA suggest that nonpharmacological therapies are better than pharmacological therapies. In the future, there is a need to include more studies of high methodological quality related to comprehensive cognitive interventions to help establish a more extensive literature foundation. Researchers should shift their research interest to outcomes other than MMSE, such as ADL and Clinical Dementia Rating (CDR) so that more network meta-analyses of cognitive interventions for AD or MCI patients can be performed.

## Acknowledgments

We would like to gratefully acknowledge the help by the following authors: Chun-Hua Zhao, Deng-Juan Qian. All authors agreed to contribute to this study.

## Author contributions

J-hL conducted the database search, screened and extracted data for the meta-analysis, prepared extracted data for the procedures, and had primary responsibility in writing this article. LL performed statistical analysis and interpretation of data. H-bZ and LH contributed to the discussion and editing. YX critically revised the draft manuscript. All authors read and approved the final manuscript.

**Data curation**: Lu Lin, Hong-bo Zhang, Lei Hang.

**Formal analysis**: Jing-hong Liang.

**Investigation**: Lu Lin, Hong-bo Zhang, Lei Hang.

**Methodology**: Jing-hong Liang, Yong Xu.

**Project administration**: Yong Xu.

**Resources**: Jing-hong Liang, Hong-bo Zhang, Lei Hang.

**Software**: Jing-hong Liang, Lu Lin.

**Supervision**: Yong Xu.

**Validation**: Yong Xu, Lu Lin.

**Writing – original draft**: Jing-hong Liang.

**Writing – review & editing**: Yong Xu, Rui-xia Jia.
